# Increased dendritic cell density and altered morphology in allergic conjunctivitis

**DOI:** 10.1038/s41433-023-02426-x

**Published:** 2023-02-06

**Authors:** Zahra Tajbakhsh, Blanka Golebiowski, Fiona Stapleton, Ali Alghamdi, Paul E. Gray, Betina Altavilla, Nancy Briggs, Isabelle Jalbert

**Affiliations:** 1 School of Optometry and Vision Science, UNSW, Sydney, NSW Australia; 2 Department of Immunology and Infectious Diseases, Sydney Children’s Hospital, Sydney, NSW Australia; 3 Stats Central, Mark Wainwright Analytical Centre, UNSW, Sydney, NSW Australia

**Keywords:** Outcomes research, Immunopathogenesis

## Abstract

**Background:**

Corneal and conjunctival epithelial dendritic cells (DC) have an established role in vernal keratoconjunctivitis, however, their role in more prevalent forms of allergic eye disease remains unclear. This study evaluated corneal and conjunctival epithelial DC density, morphology, and distribution observed using in vivo confocal microscopy (IVCM) in allergic conjunctivitis.

**Methods:**

In this prospective, observational study, 66 participants (mean age 36.6 ± 12.0 years, 56% female): 33 with allergic conjunctivitis and 33 controls were recruited. IVCM was performed at the corneal centre, inferior whorl, corneal periphery, corneal limbus, and temporal bulbar conjunctiva. DC were counted and their morphology was assessed as follows: largest cell body size, presence of dendrites, and presence of long and thick dendrites. Mixed model analysis (DC density) and non-parametric tests (DC morphology) were used.

**Results:**

DC density was higher in allergic participants at all locations (*p* ≤ 0.01), (corneal centre median (IQR) 21.9 (8.7–50.9) cells/mm^2^ vs 13.1 (2.8–22.8) cells/mm^2^; periphery 37.5 (15.6-67.2) cells/mm^2^ vs 20 (9.4–32.5) cells/mm^2^; limbus 75 (60-120) cells/mm^2^ vs 58.1 (44.4–66.2) cells/mm^2^; conjunctiva 10 (0–54.4) cells/mm^2^ vs 0.6 (0–5.6) cells/mm^2^, but not at the inferior whorl 21.9 (6.2–34.4) cells/mm^2^ vs 12.5 (1.9–37.5) cells/mm^2^, *p* = 0.20. At the corneal centre, allergic participants had larger DC bodies (*p* = 0.02), a higher proportion of DC with dendrites (*p* = 0.02) and long dendrites (*p* = 0.003) compared to controls.

**Conclusions:**

Corneal and conjunctival DC density was increased, and morphology altered in allergic conjunctivitis. These findings imply that the ocular surface immune response was upregulated and support an increased antigen-capture capacity of DC in allergic conjunctivitis.

## Introduction

Ocular allergy is an inflammatory reaction of the ocular surface resulting from a hypersensitivity reaction of the ocular adnexa to environmental allergens. Seasonal and perennial allergic conjunctivitis are most common, their incidence ranging from 6–30% [1, 2]. Whilst these milder forms of allergy result in bothersome symptoms and signs, they nevertheless pose a significant societal burden [3, 4]. Understanding the pathophysiology of allergy can optimise its diagnosis and management.

Dendritic cells (DC) are initiators of the allergic immune response [5–7]. DC capture and process antigens and migrate from affected tissues (e.g., cornea) to the lymph nodes and present antigens to T cells to initiate the immune response [5, 8]. In vivo confocal microscopy (IVCM) enables observation of DC in vivo. Increased DC density and altered morphology can signal an inflammatory or immune response [9]. The role of DC in ocular allergy is not well established.

DC density is increased in vernal keratoconjunctivitis (VKC) however it has not been investigated in allergic conjunctivitis [10–12]. In vivo studies have focused on characterising central DC density with little consideration of peripheral regions or DC morphology, both of which are important factors related to DC activation and migration. Although the primary site of ocular allergy is the conjunctiva, the cornea and conjunctiva crosstalk through release of mediators and cytokines [13–15]. When inflammation is present, DC are recruited from the limbus and move to the central cornea [16, 17]. Examining many locations concurrently gives better insights into topographical variations in immune activation during allergic eye disease. Alterations of DC morphology characterise the immune response and can serve as an early marker of disease [18, 19]. Corneal DC in participants with systemic allergy had longer dendrites compared to the non-allergic group, suggesting an increased antigen-capture capacity of DC at the ocular surface [20].

The reliability and reproducibility of DC measurements using IVCM must be established. We have reported inter and intra-observer repeatability of DC morphology and intra-observer of DC density [21, 22]. However, the inter-observer repeatability of DC density at various locations has not been reported. This study aimed to evaluate the topographical distribution, density, and morphology of corneal and conjunctival DC observed with IVCM in participants with allergic conjunctivitis. A secondary aim was to assess the inter-observer repeatability of DC density measurement at various locations.

## Materials and methods

### Study design and participants

A prospective, cross-sectional observational study was conducted. The study adhered to the Declaration of Helsinki, and participants were appraised of study procedures. Informed consent was obtained prior to enrolment in the study. The Sydney Children’s Hospitals Network Human Research Ethics Committee (HREC) (2019/ETH11844) and HREC of UNSW Sydney (HC180930) approved the study.

Participants over 18 years old were recruited. Participants with or without a prior diagnosis of allergic conjunctivitis, who had current symptoms of ocular allergy or hayfever and a positive skin prick test were assigned to the allergy group. Participants without a history or diagnosis of allergic conjunctivitis, without symptoms and a negative skin prick test were included as controls. A standard skin prick test for ten common indoor and outdoor aeroallergens of the Sydney region was conducted (Stallergenes, Antony, France, and Inmunotek, alergie e immunologia, Spain): three grass pollens, tree pollen, plant pollen, two dust mites, mould, cat, and dog dander. Participants were classified as positive if the wheal diameter was ≥3 mm for any allergen [23].

Participants were excluded if they reported use of ocular lubricants in the week prior, or current use of topical or systemic antihistamine or mast cell stabiliser or nasal corticosteroid spray or immunotherapy for aeroallergens. Other exclusion criteria included uncontrolled asthma, past anaphylactic episode, current pregnancy or breastfeeding, regular contact lens wear, ocular surface disease other than ocular allergy, Sjøgren syndrome, active intraocular inflammation, history of refractive or ocular surgery, or systemic conditions that can affect the ocular surface (e.g., diabetes, rheumatoid arthritis).

Sample size was calculated using GPower 3.1. If the controls show a mean DC density of 30 cells/mm^2^ at the corneal centre (SD = 35 cells/mm^2^) and the allergy group shows a mean density of 58 cells/mm^2^ and similar SD, there would be greater than 80% power to detect the mean difference with 27 participants per group (54 total), assuming alpha of 0.05 [22]. A 15% adjustment allowed for possible non-normal distribution in outcome variable, for a total 33 per group.

### Symptoms and signs

The Aston University Allergy Questionnaire (AUAQ), Ocular Surface Disease Index (OSDI), and Dry Eye Questionnaire (DEQ-5) were used to record ocular surface symptoms [24–26]. Non-invasive tear break-up time was assessed using a tearscope (Keeler Ltd, United Kingdom). The BHVI grading system was used to record redness [27]. Corneal epithelial disorder, bulbar conjunctival chemosis, palpebral conjunctival papillae, and follicles were graded using the Japanese grading scale [28]. The Oxford grading scale was used to grade staining [29].

### DC density and morphology

The HRT III confocal microscope with Rostock Corneal Module (Heidelberg Engineering GmbH, Heidelberg, Germany) was used to assess corneal and conjunctival DC [22]. Five locations of the corneal subbasal epithelium and conjunctival epithelium including the corneal anatomical centre, inferior whorl, peripheral cornea (temporal, 1 mm inside limbus), limbal cornea (temporal), and bulbar conjunctiva (temporal, 2–3 mm from the limbus) of the right eye were scanned, in that order. The corneal subbasal epithelium was detected at 35–70 µm and conjunctival epithelium at 5–20 µm depth. Topical anaesthetic (oxybuprocaine 0.4%, Minims, Bausch & Lomb UK) and coupling gel (hypromellose 0.3%, carbomer 980 0.2%, Ciba Vision Ophthalmics, Australia) were used between the objective lens and Tomocap contact cap.

Five best-focused images overlapping by less than 20% were selected for each location and one image from the inferior whorl (due to its small area).

Bright/ hyperreflective cells at least 10 µm in size, with a linear/curvilinear cell body, with or without dendrites (both short and long), located at the subbasal corneal epithelium and distributed among nerve fibres that do/ do not cross them or conjunctival epithelium were considered DC [21]. Density was counted manually by two experienced observers (ZT, AA) masked to group assignment and image location. Mean DC number in 5 images (or in the single inferior whorl image) was reported as cells/mm^2^. The mean value of two investigators was used.

Cell body size was graded as small (10–25 µm), medium (26–40 µm), or large (>40 µm), based on the largest cell body size observed, by a single observer (ZT) [21]. The presence of any dendrites, the presence of long dendrites, and the presence of thick dendrites was also recorded. The presence of clusters of DC at the conjunctiva was also noted. Images devoid of DC were excluded.

Inter-observer repeatability of DC density was examined. Two experienced observers (ZT, AA) independently assessed DC density in images from 66 participants.

### Air quality assessment

Data were collected between July 2019 and January 2020. From October 2019 Sydney experienced wildfires which emitted extreme levels of smoke. To examine whether these confounded measurements air quality indices were accessed, including Air Quality 24-hour Index (AQI), concentration of PM10, PM 2.5, and nitrogen dioxide (NO_2_), from the New South Wales State Government Department of Planning, Industry, and Environment (https://www.dpie.nsw.gov.au/air-quality/air-quality-data-services/data-download-facility) (Supplementary Table 1).

### Statistical analysis

SPSS software (version 26; SPSS Inc.) was used. Differences in symptoms and signs between the allergic and control groups were assessed using the Independent Sample t-test or Mann–Whitney *U* test.

Where DC density was not normally distributed, values were log-transformed adding 0.2 to values of zero. A linear mixed model with a random effect for individuals was used to examine differences in (log) DC density between groups and between locations. The fixed effect and the interaction between group and location were included. The interactions of group with age, sex or air quality were included in separate models to check the effect of these variables on between-group differences in DC density. Pairwise comparisons between groups and locations were obtained. *P*-values for multiple comparisons between locations were adjusted by Holm’s step-down Bonferroni method.

Mann–Whitney *U* test (for cell body size) and Fisher’s Exact Test (for presence of dendrites) were used to assess differences in DC morphology between groups. Friedman, and Wilcoxon Singed Ranked Test (for cell body size), Cochran’s *Q* test (for presence of dendrites) were used to assess differences in DC morphology across locations and the *p*-values for pairwise comparison between locations were adjusted by Holm’s step-down Bonferroni method.

The generalised linear mixed model for ordinal and binary outcomes that we planned on using for morphology did not converge. Therefore, smaller models within each location were estimated to assess the effect of age, sex, and air quality on DC morphology, by examining the interaction of these factors with participant group.

Associations between DC and symptoms and signs were initially assessed using a univariate Spearman’s correlation coefficient (for DC density and cell body size) and a Mann–Whitney *U* test (for presence of dendrites). Symptoms and signs that were significant at *p* < 0.01 (adjusted for multiple comparisons) were added to a multivariate generalised estimating equation (linear, ordinal, and binary logistic). A backward elimination modelling approach was used, and the final model included only those signs and symptoms that were significantly associated with DC density and morphology at *p* < 0.05.

Inter-observer agreement for DC density was examined using Bland and Altman plots and the coefficient of repeatability (CoR).

*P* < 0.05 was considered statistically significant.

## Results

Sixty-six participants (range 19.0–65.0 years) completed the study: 33 in the allergic group (mean age 39.6 ± 14.5 years, 51% female) and 33 controls (mean age 33.5 ± 7.9 years, 60% female). There was no significant difference between groups in age (*p* = 0.2) or sex (*p* = 0.6). All allergic participants tested positive to at least one dust mite or pollen allergen, 55% were positive to both dust mites and pollen, 27% were positive to only dust mites and 6% were positive to pollen only (Supplementary Table 2).

### Symptoms and signs

Ocular allergy symptoms, signs, and tear film characteristics are summarised in Table 1.

**Table 1:** Summary of findings for ocular allergy symptoms and signs in 33 allergic and 33 control participants.

Variable		Allergy group *n* = 33	Control group *n* = 33	*p*-value
Ocular surface symptoms	AUAQ, total score (0-21)	5 (0–18)	0 (0–10)	***<0.001***
	Dryness (0–3)	1 (0–3)	0 (0–3)	***0.02***
	Itchiness (0–3)	1 (0–3)	0 (0–2)	<***0.001***
	Burning (0–3)	0 (0–3)	0 (0–1)	0.14
	Stinging (0–3)	0 (0–3)	0 (0–2)	***0.03***
	Watering (0–3)	1 (0–3)	0 (0–1)	***0.001***
	Redness (0–3)	1 (0–2)	0 (0–2)	<***0.001***
	A need to rub eyes (0–3)	1 (0–3)	0 (0–2)	<***0.001***
	OSDI (0-100)	15.1 (0–48.0)	10.4 (0–50.0)	0.10
	DEQ-5 (0-22)	9 (0-18)	4.5 (0–16)	***0.01***
Ocular surface signs	Limbal redness (0–4, 0.1)	3.0 (1.5–4.0)	1.5 (1.0–2.5)	***<0.001***
	Bulbar redness (0–4, 0.1)	2.4 (1.7–3.4)	1.7 (1.2–2.2)	***<0.001***
	Palpebral redness (0–4, 0.1)	2.5 (2.0–3.5)	1.5 (1.0–2.5)	***<0.001***
	Corneal epithelial disorder (0–3, 1)	0 (0–0)	0 (0–0)	0.32
	Bulbar conjunctival chemosis (0–3, 1)	1 (0–2)	0 (0–1)	***<0.001***
	Palpebral conjunctival papillae (0–3, 1)	0 (0–0)	0 (0–0)	0.32
	Palpebral conjunctival follicles (0–3, 1)	0 (0–2)	0 (0–0)	***<0.001***
	Corneal staining (0–5, 1)	0 (0–0)	0 (0–0)	0.32
	Conjunctival staining-Nasal (0–5, 1)	0 (0–2)	0 (0–1)	***0.002***
	Conjunctival staining-Temporal (0–5, 1)	1 (0–2)	0 (0–1)	***<0.001***
	Non-invasive Tear film Break-Up Time (seconds)	8.8 (5.2-17.5)	10.7 (6.0-21.0)	***0.02***

Values are reported as median (range). Ocular signs graded in integers except for limbal, bulbar, and palpebral redness which graded in 0.1 steps. Statistically significant values (*p* < 0.05) are indicated in bold/italics.

*AUAQ* Aston University Allergy Questionnaire, *OSDI* Ocular Surface Disease Index, *DEQ-5* Dry Eye Questionnaire.

Allergic participants reported more symptoms of dryness, itchiness, stinging, watering, redness, and a need to rub eyes (*p* ≤ 0.03) but not burning. The AUAQ allergy symptom score was higher in allergic participants (*p* < 0.001), as were DEQ-5 scores (*p* = 0.01). OSDI scores were not different. Limbal, bulbar, and palpebral redness, bulbar conjunctival chemosis, palpebral follicles, and conjunctival staining were higher (*p* ≤ 0.002), and tear break-up time was lower (*p* = 0.02) in allergic participants. Epithelial disorders, staining, and bulbar conjunctival papillae were graded zero in all participants.

### Topographical distribution of DC density

**Fig. 1:**
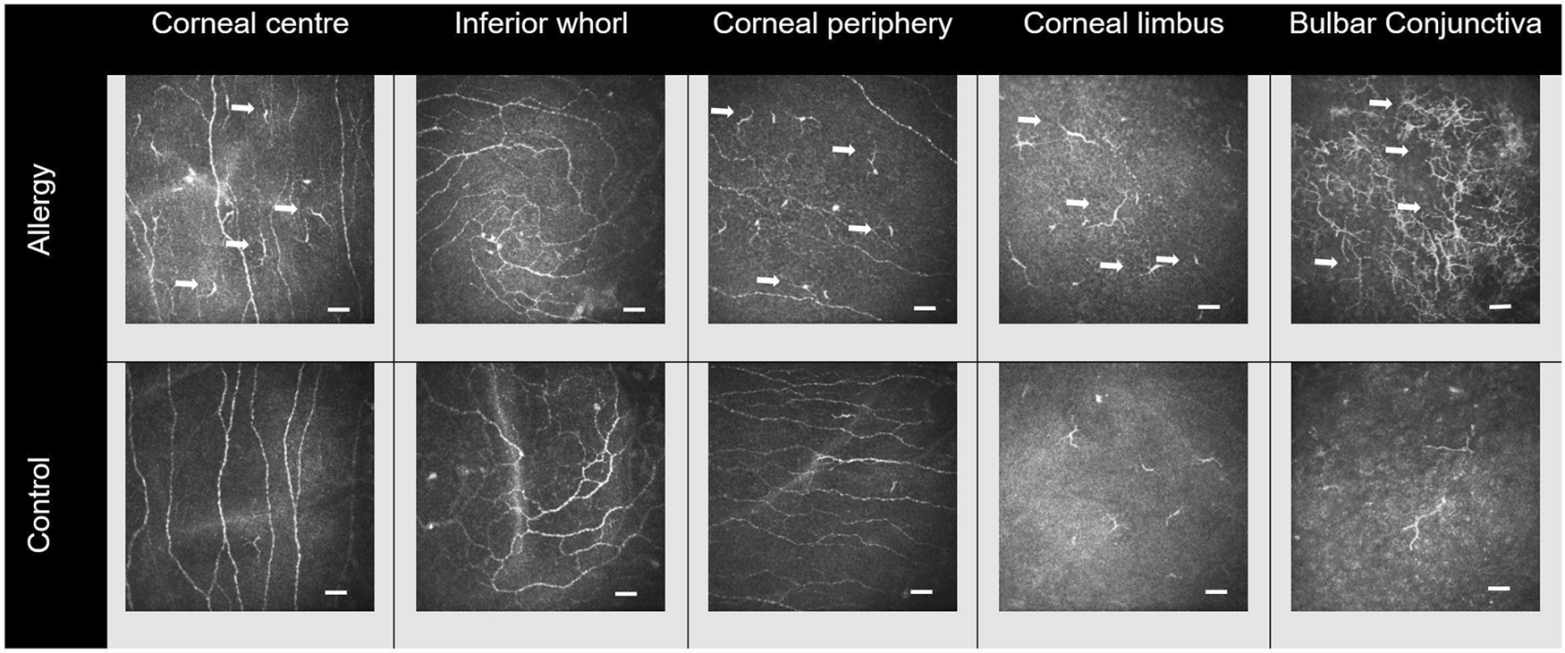
Representative images of dendritic cell (DC) at the corneal subbasal region and conjunctival epithelium in the allergy (top row) and control (bottom row) group. A higher DC density at corneal and conjunctival locations in the allergy group is evident (arrows). At the bulbar conjunctiva of allergic participants, DC tended to gather in cluster (top right). Image size = 400 × 400 µm; bar = 50 µm.

**Fig. 2:**
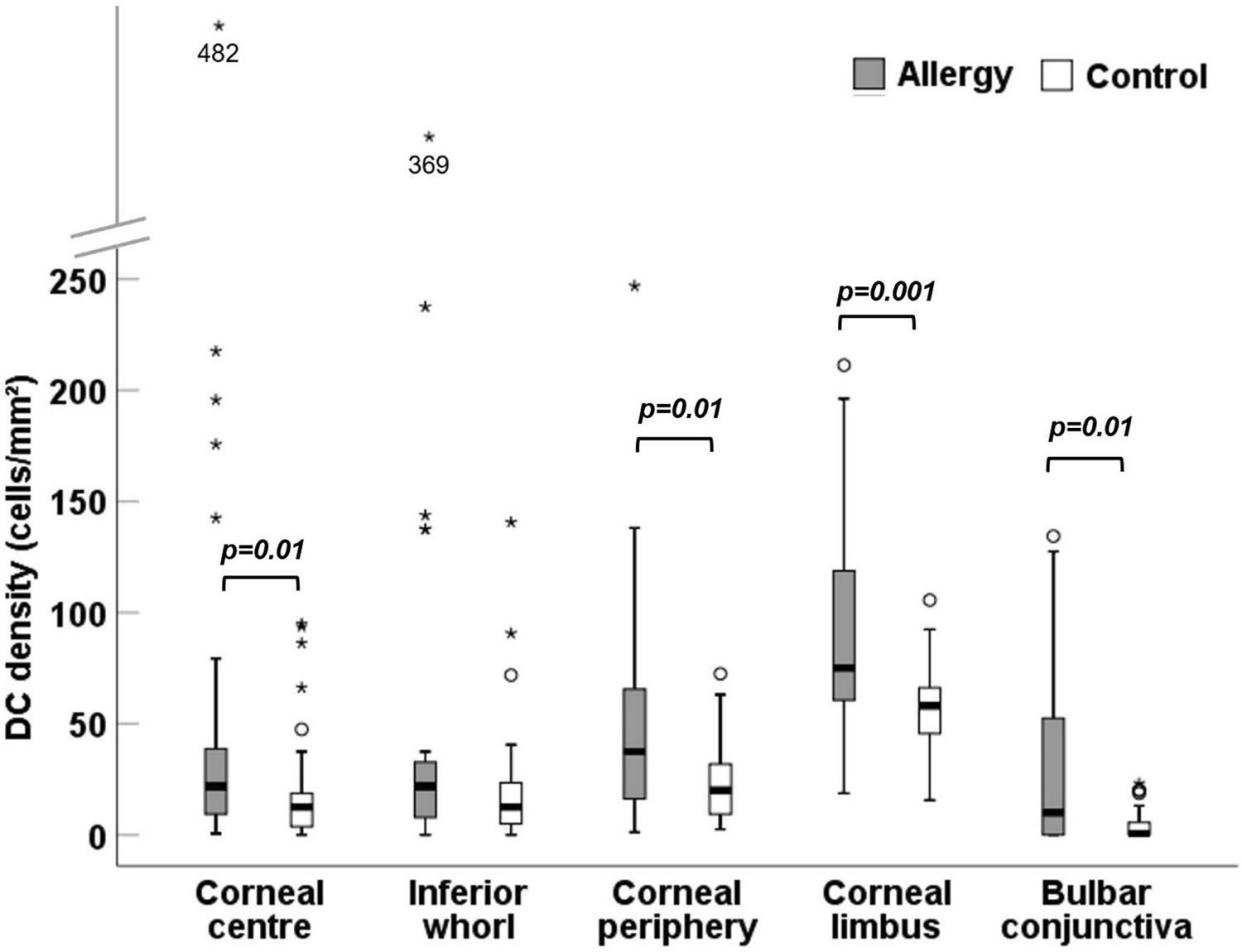
Dendritic cell (DC) density across corneal and conjunctival locations in 33 allergic and 33 control participants. Plots represent median (horizontal black line), interquartile range (box), lower and upper extremes (whiskers) and outliers lying above Q3 + 1.5*interquartile range and below Q1 − 1.5*interquartile range (circles and stars).

Representative IVCM images are shown in Fig. 1. The fixed effect of group was significant (*F* = 16.01, *p* < 0.001) showing that participant group has an impact on DC density. Simple main effects showed DC density was higher in allergic participants at all locations except at the inferior whorl (Fig. 2, Supplementary Table 3). The interactions between group and age (*p* = 0.35), sex (*p* = 0.15) or air quality (*p* ≥ 0.50) were not significant (Supplementary Table 4). Mean values for air quality indices are shown in Supplementary Table 1.

There was an effect of location on DC density (*F* = 91.92, *p* < 0.001). As the interaction between allergy group and location was not significant (*F* = 1.72, *p* = 0.15), the differences in DC density between locations were analysed together. The limbus had the highest DC density (*p* < 0.001), followed by the corneal periphery (*p* ≤ 0.01). The bulbar conjunctiva had the lowest DC density (*p* < 0.001) (Fig. 2 and Supplementary Table 3). There was no difference in DC density between the corneal centre and inferior whorl (*p* = 0.51).

### DC morphology

**Fig. 3:**
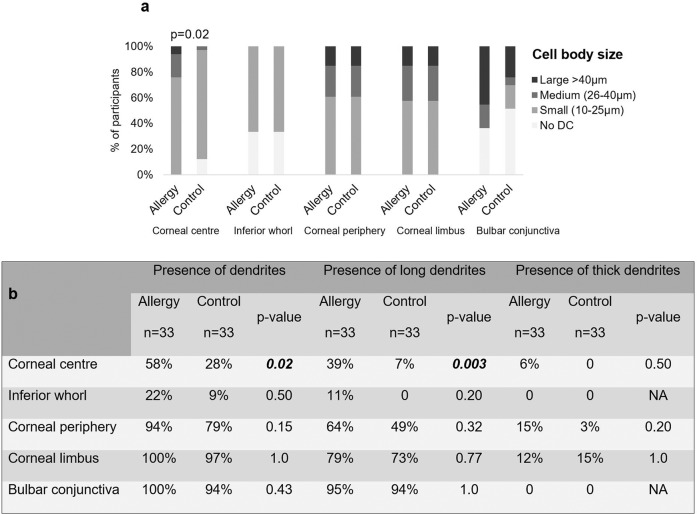
Morphology of dendritic cells at corneal and conjunctival locations in allergy and control group. Dendritic cell (DC) body size (**a**) and dendrite appearance (presence of dendrites, presence of long and thick dendrites) (**b**) at corneal and conjunctival locations in allergy (*n* = 33) and control group (*n* = 33). Dendritic cell bodies were significantly larger at the corneal centre of allergic participants compared to the control group. Images devoid of DC were excluded thus data are presented for participants with dendritic cells at each location.

DC at the corneal centre had larger cell bodies (*p* = 0.02), and a higher proportion of DC with dendrites (*p* = 0.02) and with long dendrites (*p* = 0.003) in allergic participants (Fig. 3a, b). Although the proportion of DC with dendrites and with long dendrites appeared numerically greater for allergic participants at other locations, these differences were not significant (*p* ≥ 0.15). DC cell body size was not different between allergic and non-allergic participants at other locations (*p* ≥ 0.10). The presence of thick dendrites was not different between groups (*p* ≥ 0.20). The interactions between group and age (*p* ≥ 0.06), sex (*p* ≥ 0.06) or air quality (*p* ≥ 0.08) were not significant (Supplementary Table 4).

Conjunctival DC were present in 21 (64%) participants with allergy and 16 (48%) controls. In 38% of allergic participants DC gathered in a cluster and displayed a wire-netting pattern. This was not evident in controls.

In both groups there was a difference between locations in cell body size (*p* ≤ 0.01), presence of dendrites (*p* ≤ 0.001) and presence of long dendrites (*p* ≤ 0.002). Most conjunctival DC were of the highest-grade cell body size which was significantly larger than at all corneal locations (*p* ≤ 0.001) in the allergy group. DC at the corneal periphery and limbus had larger cell body size compared to the corneal centre in controls (*p* ≤ 0.04) and compared to the inferior whorl in the allergy group (*p* ≤ 0.04) (Fig. 3a).

In both groups, most DC at the corneal periphery, limbus, and bulbar conjunctiva presented with dendrites compared to DC at the corneal centre and inferior whorl (*p* ≤ 0.04).

Both groups had a higher proportion of long dendrites at the corneal periphery, limbus, and bulbar conjunctiva compared to the inferior whorl (*p* ≤ 0.04). More cells with long dendrites were observed at the bulbar conjunctiva compared to the corneal centre (*p* ≤ 0.03). Thick dendrites were rarely observed at the corneal periphery and limbus (Fig. 3b).

### Associations between DC (density, morphology) and symptoms and signs

Eight weak correlations were observed at the univariate level (Supplementary Table 5). No associations were evident in the multivariate model. Among these, redness was the final covariate in the modelling steps with a *p*-value of 0.17.

No morphology parameter correlated with symptoms and signs (*p* ≤ 0.02, univariate level) (Supplementary Table 5).

### Inter-observer repeatability of DC density

**Fig. 4:**
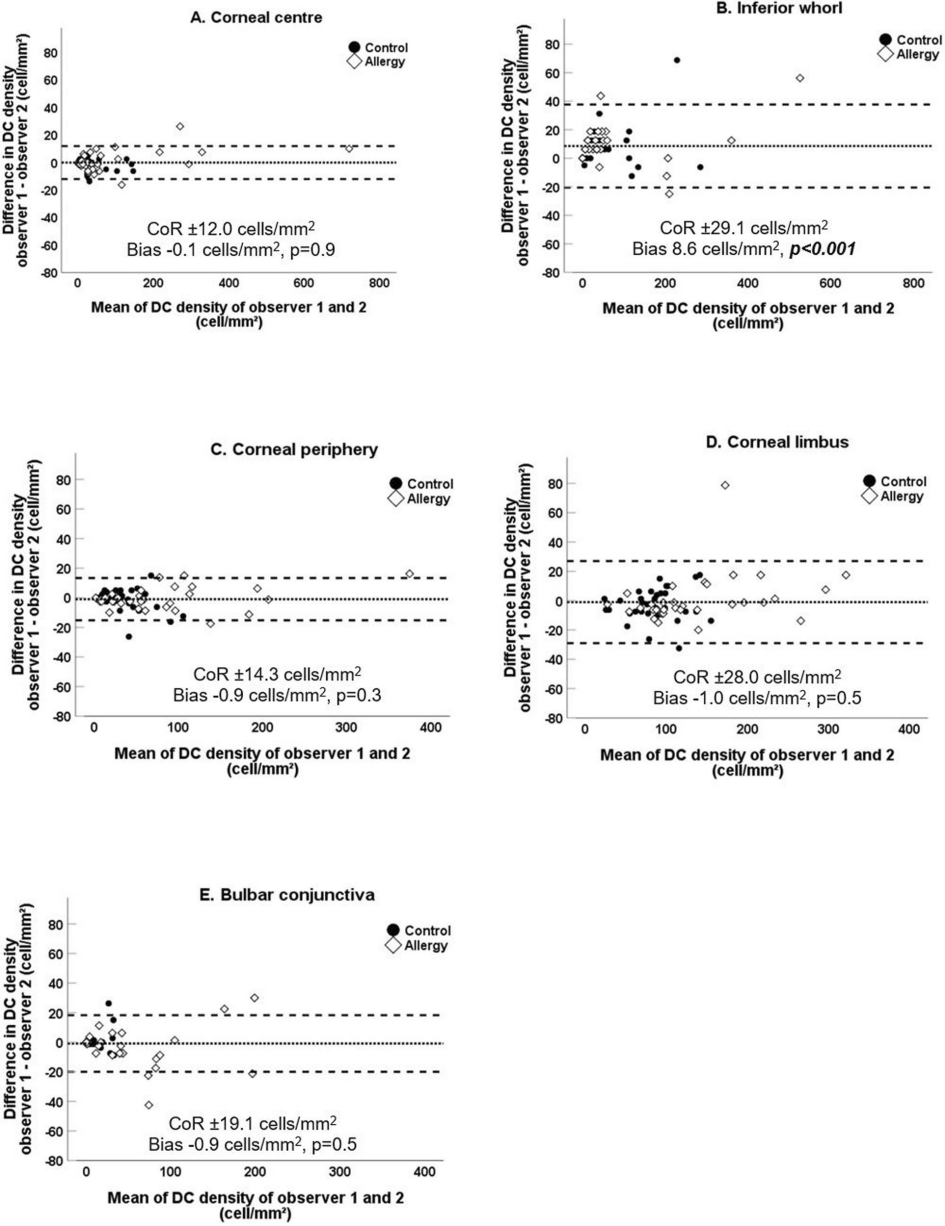
Ocular surface dendritic cell density measurements at 5 topographical locations in 66 allergic (diamond) and control (circle) participants. The difference in density measurements obtained by two observers is plotted against their means at each corneal (**A**–**D**) and conjunctival (**E**) location. Horizontal lines indicate the bias (central line) and the limits of agreement (upper and lower lines).

Figure 4 plots the differences in DC density between two observers against the mean of two measurements. The CoR of ±12.0 cells/mm^2^ at the corneal centre and the limits of agreement indicate that 95% of the differences between two observers can be expected to lie between +11.9 and −12.1 cells/mm^2^. The CoR was similar for the corneal periphery at ±14.3 cells/mm^2^, with relatively higher CoR values of ±29.1 cells/mm^2^ and ±28 cells/mm^2^ at the inferior whorl and limbus. The CoR was ±19.1 cells/mm^2^ for the conjunctiva. There was no bias between density measurements of two observers at corneal centre, periphery, limbus, or conjunctiva (*p* ≥ 0.3). The measurements of observer 1 were higher than observer 2 at the inferior whorl (*p* < 0.001) (Supplementary Table 6).

## Discussion

DC density, morphology, and distribution were assessed in vivo, as a marker of allergic conjunctivitis. Corneal and conjunctival DC density increased during allergic conjunctivitis. DC at the corneal centre had larger cell bodies, and a higher proportion of DC had dendrites and long dendrites.

DC density was higher with allergic conjunctivitis compared to controls, however the difference was not as high as that previously reported between VKC and controls [10–12]. This is the first comprehensive report of DC density at different locations including bulbar conjunctiva in allergic conjunctivitis. In an IVCM study of patients with VKC, there was a higher DC density at the corneal centre (137.4 ± 27.0 cells/mm^2^) and limbus (148.8 ± 26.0 cells/mm^2^) compared to controls (24.1 ± 10.9 cells/mm^2^ and 98.2 ± 22.6 cells/mm^2^ respectively) [12]. A higher DC density has also been reported at the peripheral cornea (VKC; 403.3 ± 106.5; normal:83.3 ± 19.0 cells/mm^2^), and at the bulbar (VKC:244.0 ± 59.7; normal:18.0 ± 5.1 cells/mm^2^) and tarsal conjunctiva (VKC:232.2 ± 145.2; normal:0 cells/mm^2^) of patients with VKC compared to controls [10, 11]. These findings support a role for DC in the pathogenesis of ocular allergy and raise the possibility that changes in DC density and morphology could serve as biomarkers for allergic eye disease, its type and severity. Investigations that would include comparisons between all forms of allergic eye disease in a single study are warranted to confirm this.

DC morphology was altered at the corneal centre of allergic participants where cell bodies were larger, and a greater proportion of DC had dendrites and long dendrites. The study was not sufficiently powered to confirm similar morphological differences observed at other locations. A study on mice [18], and human IVCM studies found similar changes in DC morphology characterised by increased DC size or DC with more processes at the corneal centre of inflamed eyes but not at the corneal periphery [30, 31].

In 38% of allergic participants, a wire-netting pattern in DC morphology at the bulbar conjunctiva was observed. A similar finding was reported in VKC patients and this suggests conjunctival inflammation is present [11].

Morphological changes to DC have previously been reported during inflammation, including an increase in DC size, cell body size, length, thickness and number of dendrites, field area and cell area of dendrites [18, 19, 32–35]. In this study, changes in DC morphology characterised by a higher proportion of DC with dendrites and with long dendrites were observed at corneal locations of allergic participants, although significant changes were found only at the corneal centre. We previously showed a higher grade of DC morphology and DC with longer dendrites at the corneal centre of participants with systemic allergy (skin prick test positive) [20] probably related to their activation status and immune response. Activation of DC in vivo can only be determined based on their morphology as information about activation markers cannot be obtained in live human studies. However, evidence from immunohistochemistry studies supports use of DC morphological alteration as a hallmark of their activation [36].

Allergic immunity is dependent on the T cell response (Th2 cells, in particular) and T cell activation requires the interaction of T cell receptor with the major histocompatibility complex (MHC) class II on DC [37, 38]. An increased density of CD11c+ MHCII+ DC were observed in the nasal epithelium of allergic rhinitis patients sensitised to house dust mite [37]. Lung DC also play an essential role in the activation of Th2 cells and the development of allergic asthma [39]. A mice model of allergic conjunctivitis revealed the important role of ocular surface DC in triggering allergic immune responses and the therapeutic benefit of DC inhibition [40]. This study aligns with these models by confirming higher DC density and altered morphology in allergic conjunctivitis.

A gradient density from high DC density at the limbus to low density at the corneal centre and inferior whorl was observed. This aligns with previously reported in vivo findings in normal and inflamed corneas and ex vivo studies [11, 12, 22, 36, 41–44]. DC have been observed to move from the limbus to the corneal centre in porcine eyes in response to contact lens wear, and in mice following bone marrow cell transplantation [16, 17]. The bulbar conjunctiva had the lowest DC density compared to corneal locations in both groups. This aligns with previous reports [11, 22, 42] and animal work [45].

The distribution of DC morphology was different between locations for cell body size, presence of dendrites and presence of long dendrites with larger cell bodies and more cells with long dendrites at the corneal periphery, limbus, and bulbar conjunctiva compared to the corneal centre and inferior whorl in both groups. This distribution is suggestive of a higher antigen-capture capacity at the corneal periphery, limbus, and bulbar conjunctiva [21, 46]. MHC class II-negative DC were predominantly found in the corneal centre and MHC class II-positive DC at the corneal periphery and limbus in mice [47]. DC with thick dendrites show higher migratory capacity [21, 41, 48]. In this study, DC with thick dendrites were observed at the corneal periphery and limbus of both groups. Due to proximity of corneal limbus to the limbal vessels, DC can migrate toward drainage lymph nodes. In the mice model plasmacytoid DC patrolled the intravascular space [45].

In this study, allergic participants had mild/moderate symptoms and signs, but higher than controls. Participants with seasonal and perennial allergic conjunctivitis were enroled, both of which cause mild symptoms and signs (commonly itching and redness), which are not sight-threatening yet can significantly interfere in daily life and detrimentally affect quality of life [49]. Weak associations (univariate level) were reported between higher DC density and many elevated symptoms and signs in the allergy group but these were not retained at the multivariate level. There may be a relationship between DC and symptoms and signs of allergy, even in this group with allergic conjunctivitis which could be explored. Further work is required to establish whether certain DC characteristics can be considered as a biomarker of ocular allergic disease.

Inter-observer repeatability for DC density was good at the corneal centre, periphery, and bulbar conjunctiva and moderate for the corneal limbus and inferior whorl. We have reported a lower intra-observer repeatability coefficient for DC density at the limbus compared to the corneal centre and bulbar conjunctiva [22]. This study supports the reliability and reproducibility of IVCM assessment of DC. Strong inter-observer agreement was previously reported for central DC density in healthy participants and those with multiple sclerosis (intra-class correlation coefficient 0.99) [50]. Correlation coefficients measure the strength of association and not the agreement between measurements. This study’s CoR is an appropriate indicator of agreement providing insights into the inherent systematic errors of measurements of DC density obtained with IVCM.

This study used IVCM to assess DC density and morphology. While IVCM allows imaging of living tissues with high resolution and contrast, exact cell phenotype cannot be confirmed. Immunohistochemistry could facilitate validation of these IVCM results. The two-dimensional nature and small field of view of IVCM images continue to limit its application.

These results highlight the potential role of corneal and conjunctival DC in ocular allergy. Augmenting knowledge of DC function in ocular allergic inflammation may help develop new therapeutic approaches.

## Summary

### What was known before

- Dendritic cells are the initiators of the allergic immune response.
- Dendritic cell density is increased in vernal keratoconjunctivitis.
- Dendritic cell density and morphology in the more common and often milder forms of allergic conjunctivitis have not been investigated.

### What this study adds

- This is the first study to assess the density, morphology, and distribution of corneal and conjunctival dendritic cells as a marker of allergic eye disease in allergic conjunctivitis using in vivo confocal microscopy Higher DC density was established at the cornea and conjunctiva in subjects with allergic conjunctivitis compared with unaffected controls.
- There was increased antigen-capture capacity of corneal dendritic cells, evidenced by larger cell bodies and a higher proportion of cells with long dendrites which was established using a novel repeatable morphology grading system.

### Supplementary information

Supplementary table 1

Supplementary table 2

Supplementary table 3

Supplementary table 4

Supplementary table 5

Supplementary table 6

## Data Availability

The datasets generated during and/or analysed during the current study are available in the Mendeley Data repository, https://data.mendeley.com/drafts/ktmb6w7yb8.
