# Supplementary material for: Increased dendritic cell density and altered morphology in allergic conjunctivitis
Source: Eye (Lond). 2023 Feb 6;37(14):2896–904. doi: 10.1038/s41433-023-02426-x (PMC10516863; doi:10.1038/s41433-023-02426-x)
Supplement: Supplementary file 1 — Supplementary table 1 [file 41433_2023_2426_MOESM1_ESM.docx]

Supplementary table 1: Mean group values for air quality indices on the day of the study visit for the duration of the data collection (July 2019 to January 2020). Values are reported as mean±SD. AQI: Air Quality 24-hour Index, PM 2.5: particulate matter particles smaller than 2.5 μm in aerodynamic diameter, PM10: particulate matter particles smaller than 10 μm in aerodynamic diameter, NO_2_: nitrogen dioxide.

|  | AQI | PM2.5 (µg/m^3^) | PM10 (µg/m^3^) | NO_2_ (pphm) |
| --- | --- | --- | --- | --- |
| Allergy participants (n=33) | 133.8±160.0 | 21.5±17.1 | 37.3±26.7 | 0.50±0.40 |
| Control participants (n=33) | 68.6±64.8 | 11.9±6.2 | 27.6±15.1 | 0.7±0.7 |
