# Supplementary material for: Increased dendritic cell density and altered morphology in allergic conjunctivitis
Source: Eye (Lond). 2023 Feb 6;37(14):2896–904. doi: 10.1038/s41433-023-02426-x (PMC10516863; doi:10.1038/s41433-023-02426-x)
Supplement: Supplementary file 2 — Supplementary table 2 [file 41433_2023_2426_MOESM2_ESM.docx]

Supplementary table 2: Skin prick test results of the 33 allergic participants. Allergen wheal size ≥ 3mm considered as skin prick test positive (+ve); DP: Dermatophagoides pteronyssinus. Grey cells indicate negative results (wheal size below 3 mm diameter).

| **Participant ID** | **Dust mite** | | **Grass Pollen** | | | **Tree Pollen** | **Plant Pollen** | **Mould** | **Animal Dander** | |
| --- | --- | --- | --- | --- | --- | --- | --- | --- | --- | --- |
|  | DP | Farinae | Rye | Bermuda | Paspalum | Cypress | Plantain | Alternaria sp | Cat | Dog |
| 1 | +ve | +ve |  |  |  |  | +ve | +ve | +ve |  |
| 2 | +ve | +ve |  | +ve | +ve |  |  |  | +ve |  |
| 3 | +ve | +ve |  | +ve | +ve |  |  |  |  |  |
| 4 |  | +ve |  |  |  | +ve |  |  |  |  |
| 5 | +ve | +ve | +ve | +ve | +ve | +ve | +ve |  | +ve | +ve |
| 6 |  | +ve |  |  | +ve |  |  |  |  |  |
| 7 | +ve | +ve | +ve | +ve | +ve | +ve | +ve |  | +ve |  |
| 8 |  | +ve |  |  |  |  |  |  |  |  |
| 9 | +ve | +ve |  | +ve |  |  |  |  |  |  |
| 10 | +ve | +ve |  |  |  |  |  |  |  |  |
| 11 | +ve | +ve |  |  | +ve |  |  |  |  |  |
| 12 | +ve | +ve |  |  |  |  |  |  |  |  |
| 13 | +ve | +ve |  |  |  |  |  |  |  |  |
| 14 | +ve | +ve |  |  |  | +ve |  |  | +ve | +ve |
| 15 |  | +ve | +ve |  |  |  |  |  |  |  |
| 16 | +ve | +ve |  |  |  |  |  |  |  |  |
| 17 |  | +ve |  |  |  |  |  |  |  |  |
| 18 | +ve | +ve |  | +ve |  |  |  | +ve |  |  |
| 19 | +ve | +ve |  |  |  |  |  |  |  |  |
| 20 | +ve | +ve | +ve | +ve | +ve | +ve | +ve | +ve | +ve | +ve |
| 21 |  |  | +ve | +ve | +ve |  |  |  |  |  |
| 22 | +ve | +ve |  |  |  |  |  |  | +ve |  |
| 23 |  |  | +ve | +ve | +ve |  |  |  |  |  |
| 24 | +ve | +ve | +ve | +ve | +ve |  | +ve | +ve |  | +ve |
| 25 | +ve | +ve | +ve | +ve |  | +ve | +ve | +ve | +ve | +ve |
| 26 | +ve | +ve |  | +ve |  |  |  |  |  |  |
| 27 |  |  | +ve | +ve |  | +ve |  |  | +ve | +ve |
| 28 |  |  | +ve | +ve | +ve | +ve | +ve | +ve |  | +ve |
| 29 | +ve | +ve |  |  |  |  |  |  |  |  |
| 30 |  |  | +ve | +ve | +ve |  | +ve | +ve |  |  |
| 31 |  | +ve | +ve | +ve | +ve |  | +ve |  |  |  |
| 32 | +ve | +ve | +ve | +ve | +ve | +ve | +ve |  |  |  |
| 33 | +ve | +ve |  |  |  |  |  |  |  |  |
