# Supplementary material for: Increased dendritic cell density and altered morphology in allergic conjunctivitis
Source: Eye (Lond). 2023 Feb 6;37(14):2896–904. doi: 10.1038/s41433-023-02426-x (PMC10516863; doi:10.1038/s41433-023-02426-x)
Supplement: Supplementary file 3 — Supplementary table 3 [file 41433_2023_2426_MOESM3_ESM.docx]

Supplementary table 3: Dendritic cell (DC) density across corneal and conjunctival locations using *in vivo* confocal microscopy in 33 allergy and 33 control participants.

| **Location** | **DC Density (cells/mm^2^) Median (IQR)** | | **p-value** |
| --- | --- | --- | --- |
|  | **Allergy**  **n=33** | **Control**  **n=33** |  |
| Corneal centre | 21.9 (8.7 – 50.9) | 13.1 (2.8 – 22.8) | ***0.01*** |
| Inferior whorl | 21.9 (6.2 – 34.4) | 12.5 (1.9 – 37.5) | 0.20 |
| Corneal periphery | 37.5 (15.6 – 67.2) | 20.0 (9.4 – 32.5) | ***0.01*** |
| Corneal limbus | 75.0 (60.0 – 120.0) | 58.1 (44.4 – 66.2) | ***0.001*** |
| Bulbar conjunctiva | 10.0 (0.0 – 54.4) | 0.6 (0.0 – 5.6) | ***0.01*** |
| p-value | ***p<0.001*** | ***p<0.001*** |  |
