# Supplementary material for: Increased dendritic cell density and altered morphology in allergic conjunctivitis
Source: Eye (Lond). 2023 Feb 6;37(14):2896–904. doi: 10.1038/s41433-023-02426-x (PMC10516863; doi:10.1038/s41433-023-02426-x)
Supplement: Supplementary file 4 — Supplementary table 4 [file 41433_2023_2426_MOESM4_ESM.docx]

Supplementary table 4: The interactions between participant group and age, sex and air quality indices for dendritic cell density and morphology. Results are summarised from multiple mixed models. Due to low occurrence of thick dendrites the interaction analysis was not performed.

|  | DC Density | DC Morphology | | | |
| --- | --- | --- | --- | --- | --- |
|  |  | Cell body size | Presence of dendrites | Presence of long dendrites | Presence of thick dendrites |
| Group*age | 0.35 | ≥0.10 | ≥0.60 | ≥0.06 | NA |
| Group*sex | 0.15 | ≥0.35 | ≥0.25 | ≥0.06 | NA |
| Group* AQI | 0.95 | ≥0.20 | ≥0.40 | ≥0.30 | NA |
| Group*PM2.5 | 0.98 | ≥0.70 | ≥0.25 | ≥0.20 | NA |
| Group* PM10 | 0.50 | ≥0.10 | ≥0.45 | ≥0.20 | NA |
| Group* NO_2_ | 0.80 | ≥0.08 | ≥0.55 | ≥0.30 | NA |
