# Supplementary material for: Increased dendritic cell density and altered morphology in allergic conjunctivitis
Source: Eye (Lond). 2023 Feb 6;37(14):2896–904. doi: 10.1038/s41433-023-02426-x (PMC10516863; doi:10.1038/s41433-023-02426-x)
Supplement: Supplementary file 5 — Supplementary table 5 [file 41433_2023_2426_MOESM5_ESM.docx]

Supplementary table 5: Association between dendritic cell density/ morphology and ocular surface symptoms/signs examined using Spearman correlation and Mann-Whitney U test (as appropriate) in all participants. All 5 locations were included; therefore, the level of significance was set at p<0.01 (adjusted for multiple comparisons).

|  | Density | DC morphology | | | |
| --- | --- | --- | --- | --- | --- |
|  |  | Cell body size | Presence of dendrites | Presence of long dendrites | Presence of thick dendrites |
| Dryness | rho=0.08  p=0.15 | rho=0.10  p=0.15 | p=0.04 | p=0.40 | p=0.15 |
| Itchiness | ***rho=0.15***  ***p=0.007*** | rho=0.13  p=0.04 | p=0.03 | p=0.15 | p=0.15 |
| Burning | rho=0.03  p=0.55 | rho=0.04  p=0.50 | p=0.90 | p=0.90 | p=0.02 |
| Stinging | rho=0.12  p=0.03 | rho=0.12  p=0.05 | p=0.15 | p=0.30 | p=0.02 |
| Watering | rho=0.02  p=0.70 | rho=0.07  p=0.25 | p=0.90 | p=0.65 | p=0.05 |
| Redness | ***rho=0.19***  ***p=0.001*** | rho=0.15  p=0.01 | p=0.03 | p=0.08 | p=0.02 |
| A need to rub eyes | rho=0.13  p=0.02 | rho=0.16  p=0.01 | p=0.02 | p=0.15 | p=0.04 |
| AUAQ, Total symptom score | rho=0.12  p=0.03 | rho=0.14  p=0.02 | p=0.03 | p=0.15 | p=0.02 |
| OSDI | rho=0.005  p=0.90 | rho=0.07  p=0.25 | p=0.80 | p=0.85 | p=0.05 |
| DEQ-5 | rho=0.05  p=0.35 | rho=0.11  p=0.06 | p=0.10 | p=0.65 | p=0.30 |
| Limbal redness | ***rho=0.19***  ***p=0.001*** | rho=0.11  p=0.06 | p=0.03 | p=0.04 | p=0.20 |
| Bulbar redness | ***rho=0.20***  ***p=0.001*** | rho=0.05  p=0.40 | p=0.10 | p=0.20 | p=0.55 |
| Palpebral redness | ***rho=0.17***  ***p=0.003*** | rho=0.04  p=0.45 | p=0.06 | p=0.15 | p=0.90 |
| Bulbar conjunctival chemosis | ***rho=0.19***  ***p=0.001*** | rho=0.10  p=0.10 | p=0.10 | p=0.15 | p=0.20 |
| Palpebral conjunctival follicle | ***rho=0.16***  ***p=0.005*** | rho=0.15  p=0.01 | p=0.30 | p=0.06 | p=0.50 |
| Conjunctival staining-Nasal | rho=0.08  p=0.15 | rho=0.07  p=0.25 | p=0.40 | p=0.70 | p=0.02 |
| Conjunctival staining-Temporal | ***rho=0.20***  ***p=0.001*** | rho=0.13  p=0.03 | p=0.02 | p=0.15 | p=0.04 |
| Non-invasive Tear film Break-Up Time | rho=-0.05  p=0.40 | rho=-0.02  p=0.75 | p=0.60 | p=0.60 | p=0.20 |
