# Supplementary material for: Increased dendritic cell density and altered morphology in allergic conjunctivitis
Source: Eye (Lond). 2023 Feb 6;37(14):2896–904. doi: 10.1038/s41433-023-02426-x (PMC10516863; doi:10.1038/s41433-023-02426-x)
Supplement: Supplementary file 6 — Supplementary table 6 [file 41433_2023_2426_MOESM6_ESM.docx]

Supplementary table 6: Corneal and conjunctival dendritic cell (DC) density from 66 participants (allergy and control) between two observers. Data presented as median (IQR; interquartile range). CoR: Coefficient of Repeatability.

| **Location** | **DC Density (cells/mm^2^) Median (IQR)** | | **Bias, cells/mm^2^,**  **(p-value)** | **CoR**  **(cells/mm^2^)** |
| --- | --- | --- | --- | --- |
|  | **Observer 1** | **Observer 2** |  |  |
| Corneal centre | 15.0 (4.7 – 35.0) | 17.5 (5.0 – 35.0) | -0.1 (0.9) | ±12.0 |
| Inferior whorl | 25.0 (6.3 – 43.8) | 12.5 (0 – 31.3) | 8.6 (***<0.001***) | ±29.1 |
| Corneal periphery | 24.4 (10.9 – 48.1) | 26.9 (12.2 – 45.3) | -0.9 (0.3) | ±14.3 |
| Corneal limbus | 63.1 (48.1 – 89.1) | 65.0 (51.3 – 88.1) | -1.0 (0.5) | ±28.0 |
| Bulbar conjunctiva | 1.8 (0.0 – 21.8) | 1.3 (0.0 – 20.0) | -0.9 (0.5) | ±19.1 |
